# Experimental nonlocality-based network diagnostics of multipartite entangled states

**DOI:** 10.1038/s41598-017-17457-0

**Published:** 2017-12-07

**Authors:** Mario A. Ciampini, Caterina Vigliar, Valeria Cimini, Stefano Paesani, Fabio Sciarrino, Andrea Crespi, Giacomo Corrielli, Roberto Osellame, Paolo Mataloni, Mauro Paternostro, Marco Barbieri

**Affiliations:** 1 Dipartimento di Fisica, Sapienza Università di Roma, P.le Aldo Moro 5, 00185 Rome, Italy; 2 Centre for Quantum Photonics, H. H. Wills Physics Laboratory and Department of Electrical and Electronic Engineering, University of Bristol, Merchant Venturers Building, Woodland Road, Bristol, BS8 1UB UK; 3 Istituto di Fotonica e Nanotecnologie - Consiglio Nazionale delle Ricerche (IFN-CNR), P.za Leonardo da Vinci, 32, I-20133 Milano, (MI) Italy; 4 Dipartimento di Fisica - Politecnico di Milano, P.za Leonardo da Vinci, 32, I-20133 Milano, (MI) Italy; 5 Centre for Theoretical Atomic, Molecular and Optical Physics, School of Mathematics and Physics, Queen’s University Belfast, Belfast, BT7 1NN United Kingdom; 6 Dipartimento di Scienze, Università degli Studi Roma Tre, Via della Vasca Navale 84, 00146 Rome, Italy

## Abstract

We introduce a novel diagnostic scheme for multipartite networks of entangled particles, aimed at assessing the quality of the gates used for the engineering of their state. Using the information gathered from a set of suitably chosen multiparticle Bell tests, we identify conditions bounding the quality of the entangled bonds among the elements of a register. We illustrate the effectiveness of our proposal by characterizing a quantum resource engineered combining two-photon hyperentanglement and photonic-chip technology. Our approach opens up future studies on medium-sized networks due to the intrinsically modular nature of cluster states, and paves the way to section-by-section analysis of larger photonics resources.

## Introduction

Quantum networks will play key roles in any embodiment of the upcoming quantum devices. They will provide distributed architectures for information processing that are able to cope with the detrimental effects of noise^1^. Moreover, they will embody versatile platforms for the simulation of complex processes and dynamics^2^. In fact, even devices that are typically conceived and considered as single, monolithic blocks, such as sensors or detectors, actually incorporate highly interconnected units, each with specialised tasks to perform. Such considerations have recently led to the proposal and demonstration of schemes for distributed quantum computing^3–9^, sensing^10^, and cryptography^11^.

The manipulation of large quantum networks requires the reliable *diagnosis* of possible imperfections at both the preparation and operation stages. In turn, information about the quality of the operations that are used to synthesize a network will be invaluable for the design of better construction stages. Recently, various strategies for the tracking of the faulty behaviour of a node or a bond in a quantum networks have been proposed. Such methods rely on statistical inference applied to quantum walk-like dynamics^12–15^.

The elements of a network might not just share a physical link, but also quantum correlations. In this case, the interest would be that of ascertaining the structure and quality of such quantum correlations. Information gathered in this respect will be key to the design of better non-classical resources. This will be very important for measurement-based one way quantum computing^16^, for instance, where the availability of high-quality entangled resources, called clauster states, is crucial to the success of any task. The relevance of such resources has prompted several experimental realisations^17–24^, some of which have highlighted their networking potential^25–27^.

In this work we propose an approach that is different from any previous one for network diagnostics considered so far^12^. The quality of quantum networks is commonly assessed by the use of the fidelity with the expected structure. This provides information about how well the generated network approximates the ideal case overall. However, it provides no details on the individual links and nodes. Here we propose a method to go beyond such limitations and that is based on two steps: first the violation of Multipartite Nonlocality Inequalities (MNLIs) is tested on different subsets of the nodes of a quantum network. Then, numerical modelling is applied to infer how much noise on each element of a specular theoretical resource should be introduced in order to predict the observed violations. We illustrate the effectiveness of the proposed approach by addressing experimentally a two-photon, four-qubit cluster state. As a suitable MNLIs, we address the inequality proposed by Werner and Wolf^28^ and, independently, Żukowski and Brukner^29^, which we dub WWZB.

## Results

### The diagnostic tool

**Figure 1:**
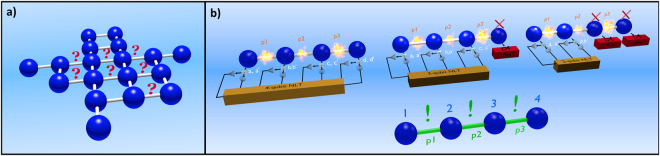
(**a**) The quality of the bonds in a cluster state with given structure needs being analysed. This amounts to assigning a number to each link that describes concisely how well that connection is established. (**b**) Our proposed strategy: a set of MNLIs is conducted on the whole cluster and to chosen subsets. From the results of such tests, we obtain a quantitative estimation of how well the connections are performed. The red crosses represent suitably chosen projective measurements (in the diagonal basis) performed on the cluster’s qubits. Such measurements either truncate the network at that location (if the qubit is on the edge of the cluster) or link adiacent nodes, so that the resulting resource can still be considered a linear cluster state but without the measured qubit.

The situation we address is illustrated in Fig. 1a. Our goal is to characterise the quality of the nodes of a given cluster state. While we assume to have full knowledge of the shape of the cluster through its adjacency matrix, we do not know *how well* the nodes are actually connected. In this sense, the problem of assessing the quality of the state is reduced to that of assigning a quality measure to each bond. The quantitative figure of merit chosen to evaluate the quality of our network is the WWZB inequality^28,29^, that we will review here for the sake of completeness.

Consider *N* agents, each addressing a single node and with the possibility to choose between two dichotomic observables $\{{\hat{A}}_{j}({{\boldsymbol{n}}}_{1}),{\hat{A}}_{j}({{\boldsymbol{n}}}_{2})\}$ (*j* = 1 … *N*), where **n**_*k*_ are local vectors in the single-qubit Bloch sphere. The observables have been rescaled so that they can only take values ±1. For local realistic theories, the correlation function for the choice of local observables is thus $E(\{{k}_{j}\})=\langle {\otimes }_{j=1}^{N}{\hat{A}}_{j}({{\bf{n}}}_{{k}_{j}})\rangle$ (*k*_*j*_ = 1, 2). By choosing a suitable function *S*({*s*_*j*_}) that can take, again, only values ±1 and depends on the indices *s*_*j*_ ∈ {−1, 1}, one can derive the following family of 4^*N*^ Bell inequalities^29^

1 $$WWZB=|\sum _{\{{s}_{j}\}=\pm 1}\,S(\{{s}_{j}\})\,\sum _{\{{k}_{j}\}=1,\,2}\,(\prod _{j=1}^{N}\,{s}_{j}^{{k}_{j}-1})\,E(\{{k}_{j}\})|\le {2}^{N},$$

whose right-hand side holds for local realistic theories. Eq. (1) contains interesting instances of Bell inequalities for *N* particles, being identical to the Clauser-Horne-Shimony-Holt version of Bell’s inequality for *N* = 2^30^. It is possible to show that the fulfilment of Eq. (1) implies the possibility to construct local realistic models for the correlation function *E*({*k*_*j*_}). The verification of such a family of inequalities is thus a necessary and sufficient condition for the local realistic description of the correlation function of an *N*-partite system^29^. In what follows, we make the choice of $S(\{{s}_{j}\})=\sqrt{2}\,\cos [\pi /4({\sum }_{j}\,{s}_{j}-N-1)]$, which allows us to recover the Mermin-Ardehali-Belinskii-Klyshko (MABK) inequality.

We have an intuition that the violation of the WWZB inequality Eq. (1) is grounded in the quality of the links within our cluster. This tests are even more informative if we consider both the whole and its subsections in which qubits are taken out by means of a measurement. We can make this connection formal by taking into account the standard procedure for forming a cluster state and establishing an appropriate noise model. First, each qubit is prepared in the superposition of its logical states $|+\rangle =(\mathrm{|0}\rangle +\mathrm{|1}\rangle )/\sqrt{2}$. Next, a controlled-Phase (C-Phase) gate is applied to each pair of nodes that need being linked. It is thus natural to assume that the quality of the link can be traced back to the quality of the C-Phase gate that has been used. This can be captured by an appropriate noise model, grounded in the processes that have governed the production of the cluster state. From the post-processing of the experimental values of WWZB inequalities [Fig. 1b] we then obtain a quantitative figure of merit providing information on the quality of each link.

Specifically, we consider the following procedure. Given the density matrix $\hat{\rho }$ describing the state of a network, we consider the noise-affected state ${\hat{\rho }}_{{\rm{fin}}}$. In general, such state will depend on a set of parameters {*p*_*j*_} that characterise the strength of the noise mechanisms under scrutiny and acting on each site and/or bond of the network itself. This can be formally expressed by considering the (trace preserving and completely positive) maps Φ such that

2 $${\hat{\rho }}_{{\rm{fin}}}={{\rm{\Phi }}}_{\{{p}_{j}\}}(\hat{\rho }\mathrm{).}$$

Notice that, for local noise models (i.e. models where the noise affects the nodes or bonds individually), ${{\rm{\Phi }}}_{\{{p}_{j}\}}$ would result from the combination of local maps, each characterised by a subset of {*p*_*j*_}. Scope of our investigation is the determination of the values $\{{p}_{j}^{\ast }\}$ that best describe the experimentally observed violation of Eq. (1), which can be quantitatively determined by minimising the distance between the theoretical prediction for *WWZB* achieved using ${\hat{\rho }}_{{\rm{fin}}}$ and the experimental values of the WWZB function.

It is important to stress that the diagnostic strategy proposed here addresses the quality of a given resource, not the actual implementation strategy chosen to accomplish this task. Therefore, our methodology can be applied *tout court* to any other resource, regardless of its implementation. In addition, in some architectures, entangling operations are implemented with high fidelity, while the state of the nodes can be corrupted by noise processes, specific of the physical system being consiodered. For instance, dissipation mechanisms (including amplitude damping), should be taken into account in atomic or atom-like systems. In photonics, the loss of quantum entanglement can be usually described in terms of pure dephasing. We have investigated the possibility of pursuing our approach in the presence of perfect gates and noisy qubits.

### Internal correlations of a 4-qubit linear cluster state

We illustrate our method in a photonic implementation, in which we realise a four-qubit linear cluster states by two-photon hyperentanglement^18,31^.

#### Experimental generation

**Figure 2:**
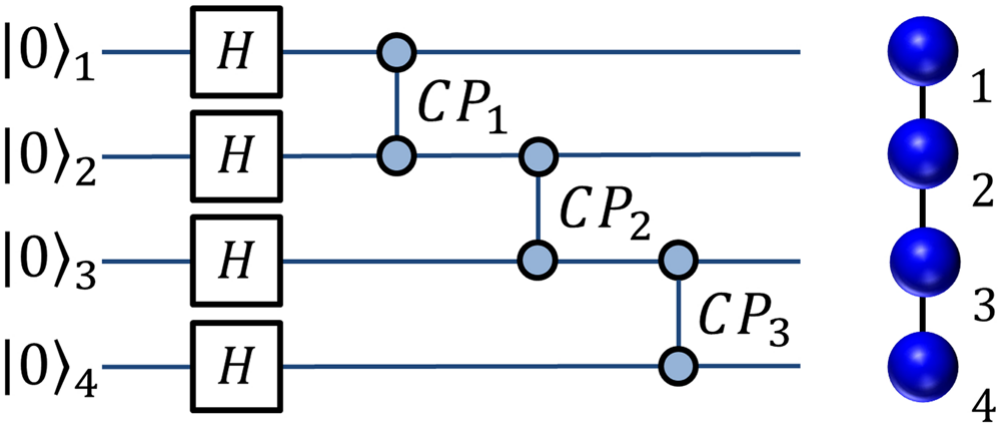
A four qubit linear cluster state can be easily obtained starting from an input |0000〉_1234_ state by rotating each qubit to the diagonal base through an Hadamard gate (H). Correlations are introduced by cascading three C-phase gates (CP) on the four initial qubits: C-PHASE_1,2_, C-PHASE_2,3_, C-PHASE_3,4_.

**Figure 3:**
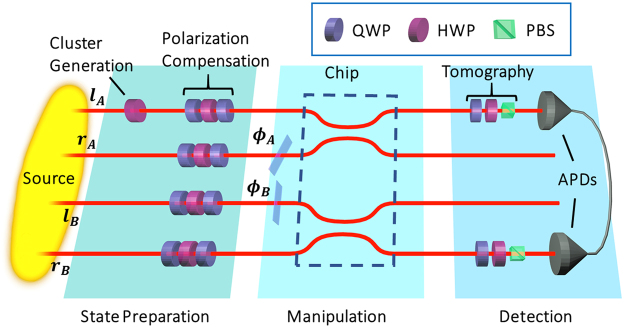
In the figure, HWP is an half waveplate, QWP is a quarter waveplate, PBS is a polarizing beamsplitter, APDs are avalanche photodiodes. The experimental setup consists of a path-polarization hyperentangled source that generates the state $|{\rm{\Xi }}\rangle =\frac{1}{2}{(|HH\rangle }_{AB}+|VV{\rangle }_{AB})\otimes {(|\ell r\rangle }_{AB}+|r\ell {\rangle }_{AB})$ ^18,34^; the source is based on the use of a 1.5-mm Beta-Barium borate (BBO) crystal within an interferometric scheme, pumped with a 100 mW laser at *λ*_*p*_ = 355 nm. Degenerate photons are produced over a filter bandwidth of 6 nm, and coupled in single mode fibres, delivering them to a femtosecond-laser written chip^31^. This requires suitable polarisation compensation of the action of the fibres on the polarisation; further, a HWP is put on the *l*_*a*_ mode in order to generate a linear cluster state by performing a C-Phase operation between polarisation and path of the same photon^18^. The chip hosts two beam-splitters that are used, in a combination with the phase retarders *ϕ*_*A*_ and *ϕ*_*B*_ to change the basis of the path qubits; polarisation analysis is performed by a standard tomographic setup. Results are obtained by measuring coincidence counts over two of the four output modes using single photon detectors. The typical counting rate through the chip was 50 coincidences/s.

The quantum circuit for the generation of such state is illustrated in Fig. 2, while the setup adopted for its experimental engineering is in Fig. 3. An hyperentangled source generates the state $|{\rm{\Xi }}\rangle =\frac{1}{2}{(|HH\rangle }_{AB}+|VV{\rangle }_{AB})\otimes {(|\ell r\rangle }_{AB}+|r\ell {\rangle }_{AB})$ with qubits encoded in the path and polarization of photons A and B. From this, we can produce a linear cluster in the form

3 $$\begin{array}{rcl}|{C}_{4}\rangle  & = & \frac{1}{2}(|{H}_{A}{H}_{B}{r}_{A}{l}_{B}\rangle +|{V}_{A}{V}_{B}{r}_{A}{l}_{B}\rangle )+\frac{1}{2}(|{H}_{A}{H}_{B}{l}_{A}{r}_{B}\rangle -|{V}_{A}{V}_{B}{l}_{A}{r}_{B}\rangle )\\  & = & \frac{1}{\sqrt{2}}{(|{\varphi }^{+}\rangle }_{AB}|{\ell }_{A}{r}_{B}\rangle +|{\varphi }^{-}{\rangle }_{AB}|{r}_{A}{\ell }_{B}\rangle ).\end{array}$$

where *H*_*x*_ (*V*_*x*_) denotes the horizontal (vertical) polarisation of photon *x* = *A*, *B*, while *r*_*x*_ (*l*_*x*_) denotes a photon taking the right (left) path. Operations on polarization-encoded qubits can easily be performed by rotating the analysis waveplates, while the two beamsplitters inside the chip and the tilting of two additional phase shifters (one for photon *a* and one for photon *b*) perform transformations on the path degree of freedom. The desired cluster state can be obtained from the hyperentangled state $|{\rm{\Xi }}\rangle$ by placing a zero-order HWP at zero degrees on mode ${\ell }_{B}$, which performs the transformation |*H*〉 → |*H*〉 and |*V*〉 → −|*V*〉. We verify that the HWP implements the correct transformation by performing polarization tomography on each of the couples $|{\ell }_{A}{r}_{B}\rangle$ and $|{r}_{A}{\ell }_{B}\rangle$. The values of the fidelities and concurrences are shown in Table 1.

**Table 1:** Values of fidelities and concurrencies observed in characterising the linear cluster state.

State	Fidelity	Concurrence
|*ϕ* ^+^〉	0.90 ± 0.04	0.81
|*ϕ* ^−^〉	0.81 ± 0.04	0.70

#### Evaluation of Multipartite Non-locality inequalities

We have performed a measurement of the four-party WWZB correlators, whose explicit form is given in the Supplementary Information. We have observed an experimental value of *WWZB* = 18.53 ± 0.23, which has to be compared with the local realistic limit 2^4^ = 16, and the maximum value that can be achieved by Quantum Mechanics $WWZ{B}_{max}=16\sqrt{2}\simeq 22.63$. The uncertainty on the value of the WWZB parameter has been calculated by propagating the errors of the experimental coincidences counts, assuming them to be Poissonian distributed. The clear deviation of the actual value from the ideal prediction flags the presence of reduced correlations within the cluster network. For the complete analysis, we have then measured WWZB correlators for different subsections of the cluster. These are shaped by means of suitable measurements on individual nodes that can take unwanted qubits out of the graph^16^.

Given a four-qubit linear cluster state it is always possible to measure a *σ*_*x*_ operator on one of the four qubits by projecting its state on one of his eigenvalues (|+〉 or |−〉). This results in a three-qubit cluster state that violates a WWZB inequality (of the MABK form) based on three qubits rather than four. The reduced three-qubit state takes, in general, different forms depending on which of the four qubits has been removed through the measurement. In turn, this implies that the WWZB parameter would take different values depending on which three-qubit cluster state is considered. In this way it is possible to study non locality properties of the following different groupings of qubits: 1-2-3, 1-2-4, 1-3-4, 2-3-4, where the qubits are ordered as (*π*_*A*_, *π*_*B*_, *k*_*A*_, *k*_*B*_) → (1, 2, 3, 4). Here, *π*_*A*,*B*_ [*k*_*A*,*B*_] stand for polarization-encoded [path-encoded] qubits.

This process can be iterated performing a second measurement on one of the three qubits remained. In this case we obtain a two-qubit entangled state, that can be tested with a two-qubit MABK inequality, which reduces to a simple Bell test (although the upper bound differs from the traditional form). The form of the resulting two-qubit state is determined by the measurements performed in order to remove two qubits from the original cluster state.

We now show that using the degree of violations of all the WWZB inequalities that can be drawn for the eleven possible four-, three- and two-qubit states, it is possible to characterize the quality of the bonds in our resource. In order to do so, we compare the experimental degree of violation achieved using a given qubit sub-grouping (such as one of those mentioned explicitly above) to what would be obtained using a theoretical resource corrupted by noise. The amount of noise that reproduces the values obtained experimentally will be used to gauge the quality of the cluster realised in the laboratory. Our results are reported in Table 2.

**Table 2:** Summary of the observed violations of the WWZB inequality for different qubit grouping within the cluster.

Qubit group	*WWZB*_*max*_	${\boldsymbol{WWZ}}{{\boldsymbol{B}}}_{{\boldsymbol{i}}}^{{\bf{\exp }}}$
1 − 2 − 4 ≡ (*π*_*A*_, *π*_*B*_, *k*_*B*_)	11.31	9.32 ± 0.19
1 − 2 − 3 ≡ (*π*_*A*_, *π*_*B*_, *k*_*A*_)	11.31	9.25 ± 0.19
1 − 3 − 4 ≡ (*π*_*A*_, *k*_*A*_, *k*_*B*_)	13.66	11.71 ± 0.17
2 − 3 − 4 ≡ (*π*_*B*_, *k*_*A*_, *k*_*B*_)	13.66	11.08 ± 0.13
1 − 4 ≡ (*π*_*A*_ − *k*_*B*_)	5.66	4.55 ± 0.13
1 − 3 ≡ (*π*_*A*_ − *k*_*A*_)	5.66	4.62 ± 0.13
2 − 3 ≡ (*π*_*B*_ − *k*_*A*_)	5.66	4.33 ± 0.15
2 − 4 ≡ (*π*_*B*_ − *k*_*B*_)	5.66	4.69 ± 0.17
1 − 2 ≡ (*π*_*A*_ − *π*_*B*_)	5.66	4.97 ± 0.14
3 − 4 ≡ (*k*_*A*_ − *k*_*B*_)	5.66	4.50 ± 0.14

#### Noise modelling

Our four-qubit cluster state can be obtained by applying a chain of three C-Phase gates to an initial ${\otimes }_{j=1}^{4}|+{\rangle }_{j}$ state of four qubits. We first model the non-ideal behaviour of the gates by allowing for their probabilitistic application according to a map of the form

4 $${M}_{ij}(\hat{\rho })={p}_{i}({\hat{U}}_{{\rm{cp}}}\,\hat{\rho }\,{\hat{U}}_{{\rm{cp}}}^{\dagger })+\mathrm{(1}-{p}_{i})\hat{\rho }.$$

Here, $\hat{\rho }={\otimes }_{j=1}^{4}|+\rangle \,\langle +{|}_{j}$ is the initial density matrix of the four qubits, ${\hat{U}}_{{\rm{cp}}}$ is the C-Phase gate between qubits *i* and *j*, and *p*_*i*_ ∈ [0, 1] is the probability that such a gate is actually applied. Eq. (4) is applied to all the three pairs of nearest-neighbour qubits of the system as

5 $${\hat{\rho }}_{{\rm{fin}}}({p}_{1},{p}_{2},{p}_{3})={M}_{34}({M}_{23}({M}_{12}({\hat{\rho }}_{in}\mathrm{))).}$$

We can thus express the resulting state as a function of the probabilities *p*_*i*_ that characterize each probabilistic map. In turn, this allows us to cast the eleven WWZB parameters as functions of the triplet (*p*_1_, *p*_2_, *p*_3_). We then look for the values $({p}_{1}^{\ast },{p}_{2}^{\ast },{p}_{3}^{\ast })$ that best describe the actual violations, by minimising the distance of the predictions to the observations. More formally, we look for the triplet of probabilities

6 $$({p}_{1}^{\ast },{p}_{2}^{\ast },{p}_{3}^{\ast })={\rm{argmin}}\,\sum _{i=1}^{11}\,|WWZ{B}_{i}({p}_{1},{p}_{2},{p}_{3})-WWZ{B}_{i}^{exp}|,$$

where the summation index runs over the 11 groupings of qubits, whose states are associated with one of the theoretical WWZB parameters *WWZB*_*i*_(*p*_1_, *p*_2_, *p*_3_) and the experimentally reconstructed one $WWZ{B}_{i}^{exp}$. Here we define ${\rm{argmin}}\,f(x)$ as the value x* in which the function f(x) achieves its minimum. The reliability of this optimisation has been tested numerically on a simulated corrupted cluster state (cf. Supplementary Information).

**Figure 4:**
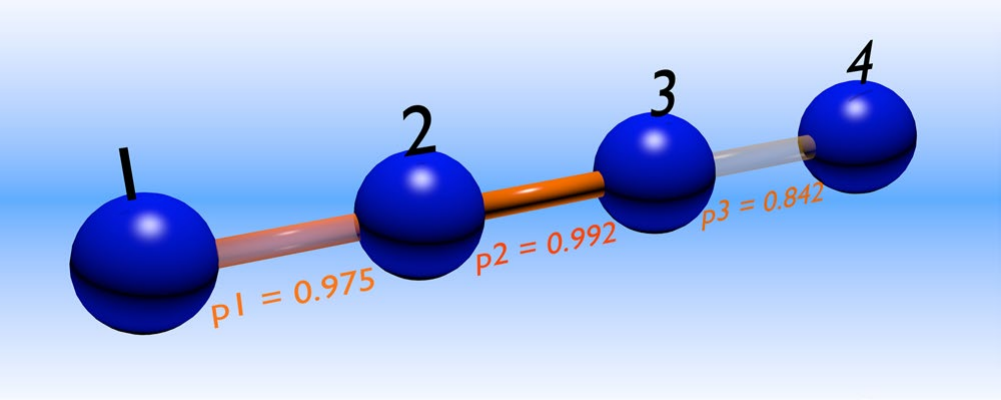
Link strength for a 4-qubit linear cluster state, using faulty-gates, each succeeding with probability *p*_*i*_. The problem of assessing the quality of the state is reduced to that of assigning a quality measure to each link. *p*_*i*_ can assume values ranging from 0 to 1: *p*_*i*_ = 0 implies full failure of the C-Phase operation in the building process of the cluster state, while *p*_*i*_ = 1 implies its full success.

The numerical minimization of the distance between theoretical model and experimentally acquired WWZB parameters leads us to ${p}_{1}^{\ast }=0.975\pm 0.024$, ${p}_{2}^{\ast }=0.992\pm 0.010$, ${p}_{3}^{\ast }=0.842\pm 0.022$ (cf. Fig. 4). These values suggest that the weakest link between the pairs of nearest-neighbour qubits is the one connecting the two path qubits, stemming from a reduced quality of the corresponding entangled resource. This observation is supported by direct experimental inspection, and it is likely due to unavoidable spatial phase instabilities present in our experimental scheme.

It could be argued that in quantum photonics systems, failures of real-world gates are seldom described by our model. A common imperfection is rather the loss of coherence, as described by single-qubit dephasing channels of the form

7 $${\varepsilon }_{j}(\hat{\rho })={q}_{j}\hat{\rho }+\mathrm{(1}-{q}_{j}){\hat{\sigma }}_{z}\hat{\rho }{\hat{\sigma }}_{z}$$

with ${\hat{\sigma }}_{z}$ the *z* Pauli matrix and *q*_*j*_ the probability that the dephasing channel does not affect qubit *j* = 1, …, 4. We can repeat our analysis by adopting such a different noise model along with perfect C-Phase gates. Direct inspection reveals that the predicted WWZB correlators only depend on the products *q*_1_ *q*_2_ and *q*_3_ *q*_4_. This is expected as, in this specific case, a dephasing channel on the qubit *j* can be replaced with an equivalent one acting on qubit *j* + 1 (*j* = 1, 3), obtaining the same theoretical expressions. A numerical optimization performed along lines analogous to those leading to Eq. (6) gives ${q}_{1}^{\ast }{q}_{2}^{\ast }=0.913\pm 0.051$, and ${q}_{3}^{\ast }{q}_{4}^{\ast }=0.892\pm 0.060$. These can be somehow interpreted as an effective strength of the nodes 2 and 3 - rather than of the links - and these values, even if statistically compatible with each other, support the previous diagnosis that path entanglement is primarily responsible for the imperfections in the whole cluster state. (Such phase instabilities are likely to be due to the presence of the fiber array that couples the free-space radiation to the chip).

Universal models are handy, but, due to their generality, they hide informative details. With minimal inspection of the physics governing the generation of our cluster state, we can obtain a more specific and refined model. As a first example, we can observe that, while the initial polarisation and path entangled states are directly produced by our source, the final cluster state is obtained by implementing a C-Phase gate between polarisation and path degrees of freedom of the same photon. As seen in Fig. 3, the cluster state |*C*_4_〉 is experimentally engineered by introducing a half-waveplate at zero degrees over mode ${\ell }_{A}$. Starting from state $|{\rm{\Xi }}\rangle$, this produces the following transformation over photon A: $|H\ell {\rangle }_{A}\to |H\ell {\rangle }_{A}$, |*Hr*〉_*A*_ → |*Hr*〉_*A*_, |*Vr*〉_*A*_ → |*Vr*〉_*A*_, $|V\ell {\rangle }_{A}\to -|V\ell {\rangle }_{A}$. This represents a C-Phase operation between the target polarization qubit and the control path qubit of photon A.

We can thus benchmark and validate our previous analysis by specializing the analysis to a form of environmental action tailored to a gate connecting polarization and path degrees of freedom. Specifically, we can use a depolarising channel of the form

8 $${\varepsilon }_{j}(\hat{\rho })={z}_{j}\hat{\rho }+\mathrm{(1}-{z}_{j})\,{\rm{diag}}(\hat{\rho })$$

to describe the corruption of the gate applied to qubits 1 and 2 (the channel occurring with a probability *z*_1_), and that applied to pair 3–4 (with corresponding probability *z*_3_), and use the probabilistic model $M(\hat{\rho })$ (with probability of occurrence *p*_2_) in order to describe the gate between qubits 2 and 3. In such case, our analysis gives the values ${z}_{1}^{\ast }=0.909\pm 0.019$, and ${z}_{3}^{\ast }=0.901\pm 0.017$ for the action of the most likely dephasing channel, and ${p}_{2}^{\ast }=0.980\pm 0.012$, once again in qualitative agreement with our previous analysis conducted under the assumption of somehow simpler noise models. This approach can be extended by including further depolarisation (captured by a probability *p*_*g*_) acting identically on every qubit as a result of traversing the chip; in this case we get: ${z}_{1}^{\ast }=0.986\pm 0.025$, ${p}_{2}^{\ast }=0.996\pm 0.007$, ${z}_{3}^{\ast }=0.967\pm 0.043$, and ${p}_{g}^{\ast }=0.866\pm 0.056$. These results are informative in the sense that depolarization can be effectively considered as acting on the state as a whole. it is significant to remark, though, that the relation between the three parameters is preserved even in this case.

We have also investigated the chance that our resource is affected by correlated noise. While the two degrees of freedom into which information is encoded in our experiment are, for all accounts, independent, in principle the possibility of correlated noise acting on the resource would not be implausible in light of the hyperentangled nature of the state. We have thus performed an additional analysis using non-local actions of the environment on the information carriers of the cluster state generated in our experiment. In particular, we have applied our procedure when the state is under the action of correlated dephasing noise built as in ref.^32^. However we have found that the distance between theoretical predictions and experimental values of the WWZB functions remains substantial under the presence of correlations in the same degree of freedom or the same photon. Similar conclusions hold for correlated amplitude damping and depolarising noise, thus demonstrating the implausibility of the conjecture of correlated noise acting on the photonic resource at hand.

## Discussion

We have experimentally assessed a diagnostic method able to probe the quality of bonds in a four-qubit cluster state implemented in a photonic circuit that blends hyperentanglement and integrated chip technology. We showed that our approach, which is based on the information gathered through the experimental violation of MNLIs and the comparison with suitably corrupted resources, allows us to identify the lowest-quality entangling operation in the state that we have engineered. We believe that our method enables a more powerful diagnosis than the simple assessment of two-qubit nonlocality tests, as it addresses general bipartitions, thus giving more information on the entanglement-sharing structure across the cluster state. Remarkably, the proposed test is run using detection tools that are exactly the same that one would use for assessing the stabilisers of the cluster, or other observables linked to the fidelity. Therefore, the only overhead come from monitoring subsections of the cluster. This cost is compensated by the increased information we get from the links, while stabiliser-based figures are averaged over the entire network.

Albeit demonstrated explicitly on a specific instance of four-partite state, the proposed method is applicable to arbitrarily connected networks of qubits, and makes no assumptions on the form of noise affecting its connections. As any diagnostics technique based on modelling, our approach would be feasible for medium-sized networks comprising a few tens of qubits, a case that will be explored in future endeavours. However, as cluster states can also be produced by fusing together shorter elements, our approach appears to be pertinent to section-by-section analysis of large photonics resources cluster, such as those built ‘just in time’ proposed in ref.^33^.

### Data availability

All data generated or analysed during this study are included in this published article (and its Supplementary Information files).

## Electronic supplementary material

Supplementary Information
