# Supplementary material for: Experimental nonlocality-based network diagnostics of multipartite entangled states
Source: Sci Rep. 2017 Dec 7;7:17122. doi: 10.1038/s41598-017-17457-0 (PMC5719363; doi:10.1038/s41598-017-17457-0)
Supplement: Supplementary file 1 — Supplementary Information [file 41598_2017_17457_MOESM1_ESM.pdf]

Supplementary Information: Experimental nonlocality-based network diagnostics of multipartite entangled states

Mario A. Ciampini[1,*], Caterina Vigliar[1], Valeria Cimini[1], Stefano Paesani[1,2], Fabio Sciarrino[1], Andrea Crespi[3,4], Giacomo Corrielli[3,4], Roberto Osellame[3,4], Paolo Mataloni[1], Mauro Paternostro[5], and Marco Barbieri[6]

[1]Dipartimento di Fisica, Sapienza Università di Roma, P.le Aldo Moro 5, 00185, Rome, Italy
[2]Centre for Quantum Photonics, H. H. Wills Physics Laboratory and Department of Electrical and Electronic Engineering, University of Bristol, Merchant Venturers Building, Woodland Road, Bristol BS8 1UB, UK.
[3]Istituto di Fotonica e Nanotecnologie - Consiglio Nazionale delle Ricerche (IFN-CNR), P.za Leonardo da Vinci, 32, I-20133 Milano (MI), Italy
[4]Dipartimento di Fisica - Politecnico di Milano, P.za Leonardo da Vinci, 32, I-20133 Milano (MI), Italy
[5]Centre for Theoretical Atomic, Molecular and Optical Physics, School of Mathematics and Physics, Queen's University Belfast, Belfast BT7 1NN, United Kingdom
[6]Dipartimento di Scienze, Università degli Studi Roma Tre, Via della Vasca Navale 84, 00146, Rome, Italy
*marioarnolfo.ciampini@uniroma1.it

ABSTRACT

We introduce a novel diagnostiic scheme for multipartite networks of entangled particles, aimed at assessing the quality of the gates used for the engineering of their state. Using the information gathered from a set of suitably chosen multiparticle Bell tests, we identify conditions bounding the quality of the entangled bonds among the elements of a register. We illustrate the effectiveness of our proposal by characterizing a quantum resource engineered combining two-photon hyperentanglement and photonic-chip technology. Our approach opens up future studies on medium-sized networks due to the intrinsically modular nature of cluster states, and paves the way to section-by-section analysis of larger photonics resources.

Theoretical background

Consider N observers each with the possibility to choose between two dichotomic observables $A_j(\mathbf{n_1})$ and $A_j(\mathbf{n_2})$, determined by some local parameters denoted by $\mathbf{n_1}$ and $\mathbf{n_2}$, which means that each local observer can choose independently two arbitrary directions. The correlation function is then the average over many runs of the experiment:

$$E(k_1,...,k_N) = \langle \prod_{j=1}^{N} A_j(\mathbf{n}_{k_j})\rangle \qquad \text{with} \qquad k_j = 1,2. \tag{S1}$$

Note that in general it is true that:

$$\sum_{s_1,...,s_N=\pm 1} S(s_1,...,s_N) \prod_{j=1}^{N} [A_j(\mathbf{n}_1) + s_j A_j(\mathbf{n}_2)] = \pm 2^N, \tag{S2}$$

where $S(s_1,...,s_N)$ stands for an arbitrary function of the summation indices $s_1,...,s_N \in \{-1,1\}$, such that its values are only ± 1. After averaging the expression over the runs of the experiment, one obtains the following set of Bell inequalities:

$$|\sum_{s_1,...,s_N=\pm 1} S(s_1,...,s_N) \sum_{k_1,...,k_N=1,2} s_1^{k_1-1}...s_N^{k_N-1} E(k_1,...,k_N)| \leq 2^N. \tag{S3}$$

In our case the function S has been chosen to be:

$$S(s_1,...,s_N) = \sqrt{2}\cos[-\frac{\pi}{4} + (s_1 + ... + s_N - N)\frac{\pi}{4}]. \tag{S4}$$

With this choice of the function S, one recovers the Mermin-Ardehali-Belinskii-Klyshko (MABK) inequalities[12], which are a particular instance of a Multipartite Nonlocality Inequality (MNLI).
Let us now derive the correlation function in the specific case of $N = 4$, i.e. the expectation value of the tensor product of four general rotations, over our state, the cluster state.
We firstly define the Pauli matrices:

$$\sigma_x = \begin{pmatrix} 0 & 1 \\ 1 & 0 \end{pmatrix}, \quad \sigma_y = \begin{pmatrix} 0 & -i \\ i & 0 \end{pmatrix}, \quad \sigma_z = \begin{pmatrix} 1 & 0 \\ 0 & -1 \end{pmatrix} \tag{S5}$$

and

$$\boldsymbol{\sigma} = (\sigma_x, \sigma_y, \sigma_z). \tag{S6}$$

A general vector in the Bloch sphere can be defined as:

$$\mathbf{n}(\theta,\phi) = (\sin(\theta)\cos(\phi), \sin(\theta)\sin(\phi), \cos(\theta)). \tag{S7}$$

A four-qubit linear cluster state can be written as:

$$\begin{aligned} |\Gamma_{\text{lin}}\rangle &= \frac{1}{2}(|0010\rangle_{1234} + |0001\rangle_{1234} - |1110\rangle_{1234} + |1101\rangle_{1234}) \\ &= \frac{1}{\sqrt{2}}(|\phi^-\rangle_{12}|10\rangle_{34} + |\phi^+\rangle_{12}|01\rangle_{34}). \end{aligned} \tag{S8}$$

which can be written in the 16-dimensional Hilbert space as:

$$|\Gamma_{\text{lin}}\rangle = \frac{1}{2}(0,1,1,0,0,0,0,0,0,0,0,0,0,-1,1,0). \tag{S9}$$

From the general form of S shown above, the correlator has the following expression:

$$\begin{aligned} &\mathrm{C}(\theta_\alpha,\phi_\alpha,\theta_\beta,\phi_\beta,\theta_\gamma,\phi_\gamma,\theta_\delta,\phi_\delta) = \\ &\qquad = \langle\Gamma_{\text{lin}}|[\mathbf{n}(\theta_\alpha,\phi_\alpha)\cdot\boldsymbol{\sigma}\otimes\mathbf{n}(\theta_\beta,\phi_\beta)\cdot\boldsymbol{\sigma}\otimes\mathbf{n}(\theta_\gamma,\phi_\gamma)\cdot\boldsymbol{\sigma}\otimes\mathbf{n}(\theta_\delta,\phi_\delta)\cdot\boldsymbol{\sigma}]|\Gamma_{lin}\rangle \\ &\qquad = -\cos(\theta_\alpha)\cos(\theta_\beta)\cos(\theta_\gamma)\cos(\theta_\delta) - \sin(\theta_\alpha)\sin(\theta_\beta)\sin(\theta_\gamma)\sin(\theta_\delta) \\ &\qquad\qquad \sin(\phi_\alpha+\phi_\beta)\sin(\phi_\gamma+\phi_\delta) \end{aligned} \tag{S10}$$

The Werner, Wolf, Zukowski and Brukner , (WWZB) parameter is composed by sixteen correlators, each for a two-setting, four-party configuration[12]:

$$\begin{aligned} \text{WWZB} = 4[&\mathrm{C}(\theta_{\alpha1},\phi_{\alpha1},\theta_{\beta1},\phi_{\beta1},\theta_{\gamma1},\phi_{\gamma1},\theta_{\delta1},\phi_{\delta1})+ \\ &+\mathrm{C}(\theta_{\alpha1},\phi_{\alpha1},\theta_{\beta1},\phi_{\beta1},\theta_{\gamma1},\phi_{\gamma1},\theta_{\delta2},\phi_{\delta2})+ \\ &+\mathrm{C}(\theta_{\alpha1},\phi_{\alpha1},\theta_{\beta1},\phi_{\beta1},\theta_{\gamma2},\phi_{\gamma2},\theta_{\delta1},\phi_{\delta1})+ \\ &-\mathrm{C}(\theta_{\alpha1},\phi_{\alpha1},\theta_{\beta1},\phi_{\beta1},\theta_{\gamma2},\phi_{\gamma2},\theta_{\delta2},\phi_{\delta2})+ \\ &+\mathrm{C}(\theta_{\alpha1},\phi_{\alpha1},\theta_{\beta2},\phi_{\beta2},\theta_{\gamma1},\phi_{\gamma1},\theta_{\delta1},\phi_{\delta1})+ \\ &-\mathrm{C}(\theta_{\alpha1},\phi_{\alpha1},\theta_{\beta2},\phi_{\beta2},\theta_{\gamma1},\phi_{\gamma1},\theta_{\delta2},\phi_{\delta2})+ \\ &-\mathrm{C}(\theta_{\alpha1},\phi_{\alpha1},\theta_{\beta2},\phi_{\beta2},\theta_{\gamma2},\phi_{\gamma2},\theta_{\delta1},\phi_{\delta1})+ \\ &-\mathrm{C}(\theta_{\alpha1},\phi_{\alpha1},\theta_{\beta2},\phi_{\beta2},\theta_{\gamma2},\phi_{\gamma2},\theta_{\delta2},\phi_{\delta2})+ \\ &+\mathrm{C}(\theta_{\alpha2},\phi_{\alpha2},\theta_{\beta1},\phi_{\beta1},\theta_{\gamma1},\phi_{\gamma1},\theta_{\delta1},\phi_{\delta1})+ \\ &-\mathrm{C}(\theta_{\alpha2},\phi_{\alpha2},\theta_{\beta1},\phi_{\beta1},\theta_{\gamma1},\phi_{\gamma1},\theta_{\delta2},\phi_{\delta2})+ \\ &-\mathrm{C}(\theta_{\alpha2},\phi_{\alpha2},\theta_{\beta1},\phi_{\beta1},\theta_{\gamma2},\phi_{\gamma2},\theta_{\delta1},\phi_{\delta1})+ \\ &-\mathrm{C}(\theta_{\alpha2},\phi_{\alpha2},\theta_{\beta1},\phi_{\beta1},\theta_{\gamma2},\phi_{\gamma2},\theta_{\delta2},\phi_{\delta2})+ \\ &-\mathrm{C}(\theta_{\alpha2},\phi_{\alpha2},\theta_{\beta2},\phi_{\beta2},\theta_{\gamma1},\phi_{\gamma1},\theta_{\delta1},\phi_{\delta1})+ \\ &-\mathrm{C}(\theta_{\alpha2},\phi_{\alpha2},\theta_{\beta2},\phi_{\beta2},\theta_{\gamma1},\phi_{\gamma1},\theta_{\delta2},\phi_{\delta2})+ \\ &-\mathrm{C}(\theta_{\alpha2},\phi_{\alpha2},\theta_{\beta2},\phi_{\beta2},\theta_{\gamma2},\phi_{\gamma2},\theta_{\delta1},\phi_{\delta1})+ \\ &+\mathrm{C}(\theta_{\alpha2},\phi_{\alpha2},\theta_{\beta2},\phi_{\beta2},\theta_{\gamma2},\phi_{\gamma2},\theta_{\delta2},\phi_{\delta2})]. \end{aligned} \tag{S11}$$

Given the dimension N=4 of our state, the bound imposed by local realistic theories becomes $|WWZB| < 16$. In order to get a violation, we find the angles that maximize the value of WWZB:

$$\begin{aligned}
&\theta_{\alpha 1} = \frac{\pi}{2}, \quad \theta_{\alpha 2} = \frac{\pi}{2}, \quad \theta_{\beta 1} = \frac{\pi}{2}, \quad \theta_{\beta 2} = \frac{\pi}{2}, \\
&\theta_{\gamma 1} = \frac{\pi}{2}, \quad \theta_{\gamma 2} = \frac{\pi}{2}, \quad \theta_{\delta 1} = \frac{\pi}{2}, \quad \theta_{\delta 2} = \frac{\pi}{2} \\
&\phi_{\alpha 1} = \frac{5\pi}{4}, \quad \phi_{\alpha 2} = \frac{3\pi}{4}, \quad \phi_{\beta 1} = 0, \quad \phi_{\beta 2} = -\frac{\pi}{2}, \\
&\phi_{\gamma 1} = 0, \quad \phi_{\gamma 2} = \frac{\pi}{2}, \quad \phi_{\delta 1} = 0, \quad \phi_{\delta 2} = \frac{\pi}{2}
\end{aligned} \tag{S12}$$

Notice that all the θ's angles are $\frac{\pi}{2}$. This means that the all the observables used in the WWZB inequality for the cluster state are off-diagonal:

$$\begin{aligned}
r_{\alpha 1} &= -J = -(\sigma_x + \sigma_y) = \begin{pmatrix} 0 & -\frac{1-i}{\sqrt{2}} \\ -\frac{1+i}{\sqrt{2}} & 0 \end{pmatrix}, \\
r_{\beta 1} &= X = \sigma_x = \begin{pmatrix} 0 & 1 \\ 1 & 0 \end{pmatrix}, \\
r_{\gamma 1} &= X = \sigma_x = \begin{pmatrix} 0 & 1 \\ 1 & 0 \end{pmatrix}, \\
r_{\delta 1} &= X = \sigma_x = \begin{pmatrix} 0 & 1 \\ 1 & 0 \end{pmatrix}, \\
r_{\alpha 2} &= -K = -(\sigma_x - \sigma_y) = \begin{pmatrix} 0 & -\frac{1+i}{\sqrt{2}} \\ -\frac{1-i}{\sqrt{2}} & 0 \end{pmatrix}, \\
r_{\beta 2} &= -Y = -\sigma_y = \begin{pmatrix} 0 & i \\ -i & 0 \end{pmatrix}, \\
r_{\gamma 2} &= Y = \sigma_y = \begin{pmatrix} 0 & -i \\ i & 0 \end{pmatrix}, \\
r_{\delta 2} &= -Y = -\sigma_y = \begin{pmatrix} 0 & i \\ -i & 0 \end{pmatrix}.
\end{aligned} \tag{S13}$$

Each term in the WWZB parameter, with the angles chosen beforehand, contributes to the violation of the local realistic constraint by a certain amount, that we report in the column labeled c_{teo} of Table S.I, after renaming the correlators as in the following:

$$\begin{aligned}
c_1 &= \mathrm{C}(\theta_{\alpha 1}, \phi_{\alpha 1}, \theta_{\beta 1}, \phi_{\beta 1}, \theta_{\gamma 1}, \phi_{\gamma 1}, \theta_{\delta 1}, \phi_{\delta 1}) \\
c_2 &= \mathrm{C}(\theta_{\alpha 1}, \phi_{\alpha 1}, \theta_{\beta 1}, \phi_{\beta 1}, \theta_{\gamma 1}, \phi_{\gamma 1}, \theta_{\delta 2}, \phi_{\delta 2}) \\
c_3 &= \mathrm{C}(\theta_{\alpha 1}, \phi_{\alpha 1}, \theta_{\beta 1}, \phi_{\beta 1}, \theta_{\gamma 2}, \phi_{\gamma 2}, \theta_{\delta 1}, \phi_{\delta 1}) \\
c_4 &= \mathrm{C}(\theta_{\alpha 1}, \phi_{\alpha 1}, \theta_{\beta 1}, \phi_{\beta 1}, \theta_{\gamma 2}, \phi_{\gamma 2}, \theta_{\delta 2}, \phi_{\delta 2}) \\
c_5 &= \mathrm{C}(\theta_{\alpha 1}, \phi_{\alpha 1}, \theta_{\beta 2}, \phi_{\beta 2}, \theta_{\gamma 1}, \phi_{\gamma 1}, \theta_{\delta 1}, \phi_{\delta 1}) \\
c_6 &= \mathrm{C}(\theta_{\alpha 1}, \phi_{\alpha 1}, \theta_{\beta 2}, \phi_{\beta 2}, \theta_{\gamma 1}, \phi_{\gamma 1}, \theta_{\delta 2}, \phi_{\delta 2}) \\
c_7 &= \mathrm{C}(\theta_{\alpha 1}, \phi_{\alpha 1}, \theta_{\beta 2}, \phi_{\beta 2}, \theta_{\gamma 2}, \phi_{\gamma 2}, \theta_{\delta 1}, \phi_{\delta 1}) \\
c_8 &= \mathrm{C}(\theta_{\alpha 1}, \phi_{\alpha 1}, \theta_{\beta 2}, \phi_{\beta 2}, \theta_{\gamma 2}, \phi_{\gamma 2}, \theta_{\delta 2}, \phi_{\delta 2}) \\
c_9 &= \mathrm{C}(\theta_{\alpha 2}, \phi_{\alpha 2}, \theta_{\beta 1}, \phi_{\beta 1}, \theta_{\gamma 1}, \phi_{\gamma 1}, \theta_{\delta 1}, \phi_{\delta 1}) \\
c_{10} &= \mathrm{C}(\theta_{\alpha 2}, \phi_{\alpha 2}, \theta_{\beta 1}, \phi_{\beta 1}, \theta_{\gamma 1}, \phi_{\gamma 1}, \theta_{\delta 2}, \phi_{\delta 2}) \\
c_{11} &= \mathrm{C}(\theta_{\alpha 2}, \phi_{\alpha 2}, \theta_{\beta 1}, \phi_{\beta 1}, \theta_{\gamma 2}, \phi_{\gamma 2}, \theta_{\delta 1}, \phi_{\delta 1}) \\
c_{12} &= \mathrm{C}(\theta_{\alpha 2}, \phi_{\alpha 2}, \theta_{\beta 1}, \phi_{\beta 1}, \theta_{\gamma 2}, \phi_{\gamma 2}, \theta_{\delta 2}, \phi_{\delta 2})
\end{aligned}$$

Correlators	Operator	c_{teo}	c_{exp}
c_1	-JXXX	0	-0.09 ± 0.01
c_2	JXXY	0.71	0.40 ± 0.02
c_3	-JXYX	0.71	0.46 ± 0.02
c_4	JXYY	0	0.04 ± 0.02
c_5	JYXX	0	0.19 ± 0.01
c_6	-JYXY	-0.71	-0.66 ± 0.01
c_7	JYYX	-0.71	-0.66 ± 0.01
c_8	-JYYY	0	0.01 ± 0.01
c_9	-KXXX	0	0.12 ± 0.01
c_{10}	KXXY	-0.71	-0.74 ± 0.01
c_{11}	-KXYX	-0.71	-0.74 ± 0.01
c_{12}	KXYY	0	-0.002 ± 0.015
c_{13}	KYXX	0	0.16 ± 0.01
c_{14}	-KYXY	-0.71	-0.49 ± 0.01
c_{15}	KYYX	-0.71	-0.48 ± 0.02
c_{16}	-KYYY	0	-0.02 ± 0.02

Table S.I. Contribution of each correlator c_i to the $WWZB_4$ parameter defined in Eq.S11. In the second column, the corresponding operator to perform to the linear cluster state. c_{teo} is the theoretical expected value, c_{exp} is the experimental result.

$$c_{13} = \mathrm{C}(\theta_{\alpha 2}, \phi_{\alpha 2}, \theta_{\beta 2}, \phi_{\beta 2}, \theta_{\gamma 1}, \phi_{\gamma 1}, \theta_{\delta 1}, \phi_{\delta 1})$$
$$c_{14} = \mathrm{C}(\theta_{\alpha 2}, \phi_{\alpha 2}, \theta_{\beta 2}, \phi_{\beta 2}, \theta_{\gamma 1}, \phi_{\gamma 1}, \theta_{\delta 2}, \phi_{\delta 2})$$
$$c_{15} = \mathrm{C}(\theta_{\alpha 2}, \phi_{\alpha 2}, \theta_{\beta 2}, \phi_{\beta 2}, \theta_{\gamma 2}, \phi_{\gamma 2}, \theta_{\delta 1}, \phi_{\delta 1})$$
$$c_{16} = \mathrm{C}(\theta_{\alpha 2}, \phi_{\alpha 2}, \theta_{\beta 2}, \phi_{\beta 2}, \theta_{\gamma 2}, \phi_{\gamma 2}, \theta_{\delta 2}, \phi_{\delta 2})$$

Therefore, in order to obtain all the sixteen correlators given above, we need to perform the 4-qubit operators shown in Table S.I.

Each observable for the single qubit is in the form: $\begin{pmatrix} 0 & e^{i\phi} \\ e^{-i\phi} & 0 \end{pmatrix}$ with a suitable choice of the phase. Therefore its measurement amounts to projecting its eigenvectors $(-e^{i\phi}, 1)$ and $(e^{i\phi}, 1)$.

For instance, taking $\phi = \frac{\pi}{4}$, the eigenvectors of J are:

$$\mathbf{q} = (\frac{1-i}{\sqrt{2}}, 1) \qquad \text{and} \qquad \mathbf{q_1} = (-\frac{1-i}{\sqrt{2}}, 1)$$

with eigenvalues $\frac{1}{\sqrt{2}}(1, -1)$.

Performing a projection on these eigenvectors means finding the unitary transformation that rotates the computational basis into the rotated basis of these eigenvectors, and then invert it. This operation can be implemented by a combination of a HWP and a QWP:

$$U = HWP(\frac{\pi}{8} + \frac{\phi}{4}) QWP(\frac{\phi}{2}). \tag{S14}$$

The inverse is then easily derived by noting that, if we use $\phi = \frac{\pi}{4}$:

$$HWP(\frac{\pi}{16}) QWP(\frac{\pi}{4}) \mathbf{q} = (1, 0), \tag{S15}$$
$$HWP(\frac{\pi}{16} + \frac{\pi}{4}) QWP(\frac{\pi}{4}) \mathbf{q}_1 = (1, 0).$$

In this way we can perform the projection on the rotated basis of the eigenvectors of J and K, while for X and Y we can use the standard tomographic settings. The same applies for the observables in the path degree of freedom: a phase shifter and a beamsplitter, allowing to measure all observables in the form we need.

State	Fidelity	Concurrence
$\lvert\phi^+\rangle_{r_B\ell_A}$	0.90 ± 0.04	0.81
$\lvert\phi^+\rangle_{r_A\ell_B}$	0.87 ± 0.04	0.75

Table S.II. Values of fidelities and concurrencies observed in characterizing the HE state via polarization tomographies.

Experimental realization

The experimental setup

The experimental setup consists of an hyperentanglement (HE) photon state source, a manipulation stage (integrated photonic circuit) and a detection apparatus.

In this work we use both path and polarization degrees of freedom to build a 2-photon 4-qubit linear cluster state from an hyperentangled photon state source. This source generates two photons, entangled both in path and polarization degrees of freedom:

$$|\Omega_{HE}\rangle = \frac{1}{\sqrt{2}}(|H_AH_B\rangle + |V_AV_B\rangle)\otimes\frac{1}{\sqrt{2}}(|r_A\ell_B\rangle + |\ell_Ar_B\rangle) \tag{S16}$$

where A and B label the two photons, r_A, ℓ_A, r_B, ℓ_B label the path modes and H, V label the two possible polarizations for each photon. Each of the four spatial modes can be manipulated independently by custom-made half mirrors, half lenses and a single mode fiber array, with four GRIN lenses at the input side. The output side of the fiber array is coupled to a glass chip, manufactured with the femtosecond laser writing tecnique: it consists of two directional couplers, mainly two beam splitters (BSs). r_A and ℓ_A are coupled on the first BS, while r_B and ℓ_B on the second. This integrated quantum circuit preserves polarization and, when thermically insulated, grants phase stability thus allowing to perform arbitrary basic operations on path-encoded qubits, without compromising the polarization encoding.

Besides, polarization compensation has been performed individually for each of the four modes with a set of two QWPs and a HWP, by which it is possible to compensate any arbitrary rotation of the polarization due to fibers and chip. The output of the chip is collected by a 10x objective and then coupled to the detectors through multi mode fibers, in order to measure coincidences between the output modes r'_A and ℓ'_B (two of the outputs of the two BSs inside the chip). To ensure the photon indistinguishability two interference filters centred at 710 nm select a 5 nm bandwidth. Passing through a set of HWP, QWP and PBSs the photons are analyzed in polarization. In this way we are able to characterize the HE state by performing a complete two-qubit tomography on each of the couples $|\ell_Ar_B\rangle$ and $|r_A\ell_B\rangle$. The tomography of the states are reported in Fig.S1. Fidelity and concurrence of such tomographies are reported in the table S.II.

Building the cluster state

Starting from the HE state, we engineered the four-qubit linear cluster state, encoded in path and polarization degrees of freedom, as shown above in Eq.S8:

$$\begin{aligned}|\Gamma_{\text{lin}}\rangle &= \frac{1}{2}(|0010\rangle_{1234} + |0001\rangle_{1234} - |1110\rangle_{1234} + |1101\rangle_{1234}) = \\ &= \frac{1}{2}(|H_AH_Br_A\ell_B\rangle + |H_AH_B\ell_Ar_B\rangle - |V_AV_Br_A\ell_B\rangle + |V_AV_B\ell_Ar_B\rangle) = \\ &= \frac{1}{\sqrt{2}}(|\phi^+\rangle|\ell_Ar_B\rangle + |\phi^-\rangle|r_A\ell_B\rangle).\end{aligned} \tag{S17}$$

The desired cluster state can be obtained from the hyperentangled state by placing a zero-order HWP at zero degrees on mode ℓ_A, which performs the transformation: $|\ell_AH\rangle \to |\ell_AH\rangle$ and $|\ell_AV\rangle \to -|\ell_AV\rangle$. We verify that the HWP implements the correct transformation by performing polarization tomography on projecting on each of the path pairs $|\ell_Ar_B\rangle$ and $|r_A\ell_B\rangle$. The results are reported in Fig.S2, along with the values of the fidelities and concurrences shown in Table S.III.

The WWZB parameter

As we mentioned above, operations on polarization-encoded qubits can be easily performed by rotating the analysis waveplates, while the two beam splitters inside the chip and the tilting of two additional phase shifters (one for photon A and one for photon B) perform transformations on path. The experimental values of each correlator c_i is reported in Tab. S.I. The correlators,

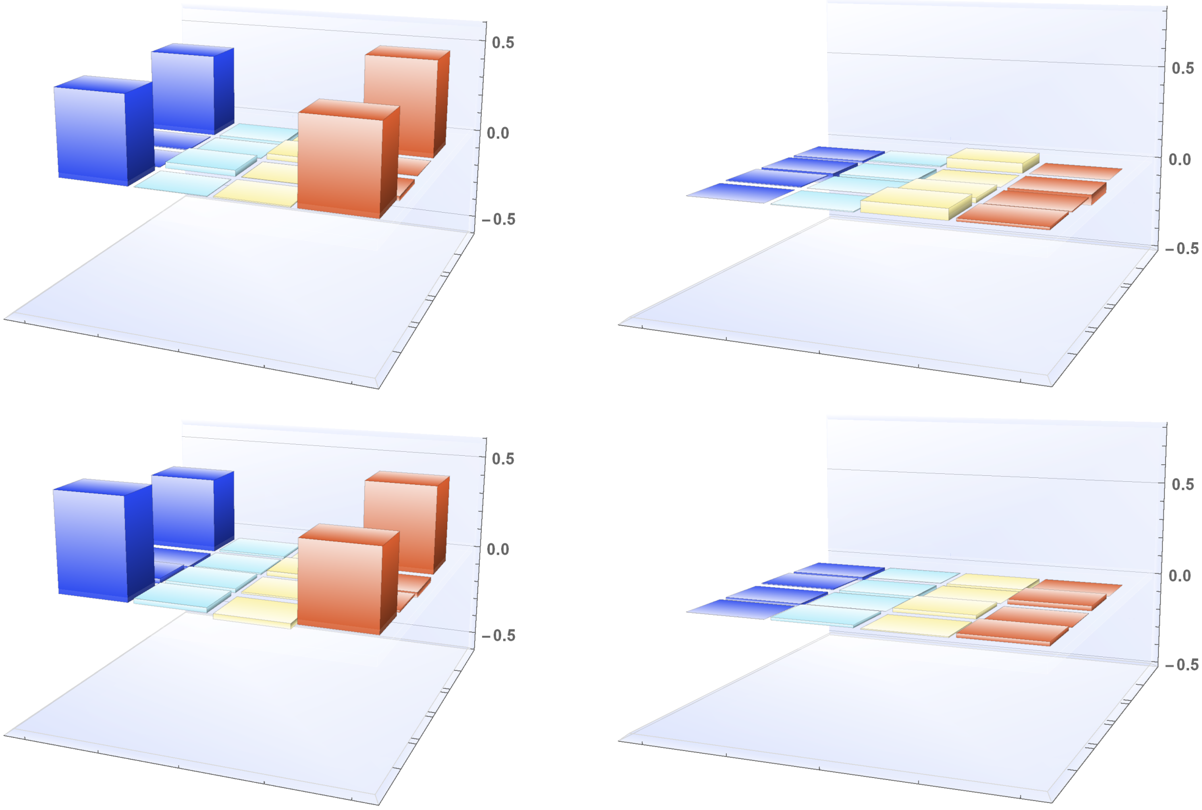

Figure S1. Reconstruction of density matrices obtained with $\theta = 0$: tomographies of $|\ell_A r_B\rangle$ and $|r_A \ell_B\rangle$ respectively (on the left side the real part, on the right side the imaginary part). As expected, they both show $|\phi^+\rangle$ behaviour.

State	Fidelity	Concurrence
$\vert\phi^+\rangle$	0.90 ± 0.04	0.81
$\vert\phi^-\rangle$	0.81 ± 0.04	0.70

Table S.III. Values of fidelities and concurrencies observed in characterizing the linear cluster state.

summed as in Eq. S12, give the experimental WWZB parameter for our resource:

$$WWZB = 18.53 \pm 0.23, \tag{S18}$$

where the error has been calculated by propagating the errors of the coincidences counts, assuming them to be Poissonian distributed. The value obtained is 11 standard deviations away from the bound of local realistic theories.

Non Locality based assessments of multipartite quantum resources

In this section we demonstrate that it is possible to infer the quality of the cluster resource by considerations on its non-locality. Given a four-qubit linear cluster state it is always possible to measure a σ_x operator on one of the four qubits, projecting its state on one of his eigenvalues ($|+\rangle$ or $|-\rangle$). The resulting three-qubit state is again a cluster state, which violates a general Mermin inequality (i.e. a WWZB parameter) that involves now three qubits instead of four. This three-qubit state is generally different depending on which of the four qubits has been crossed out by the measurement, implying that the measures that characterize the WWZB parameters to calculate can be different. In this way it is possible to study non locality properties of different groupings of the qubits: 1-2-3, 1-2-4, 1-3-4, 2-3-4, having used always the same convention for the order of the qubits (π_A, π_B, k_A, k_B), that means the first two are polarization encoded and the last two path encoded.

This process can be iterated performing a second measurement on one of the three qubits remained. In this case we obtain a two-qubit entangled state, that can be tested with a two-qubit Mermin inequality that reduces to a simple Bell test. The state is

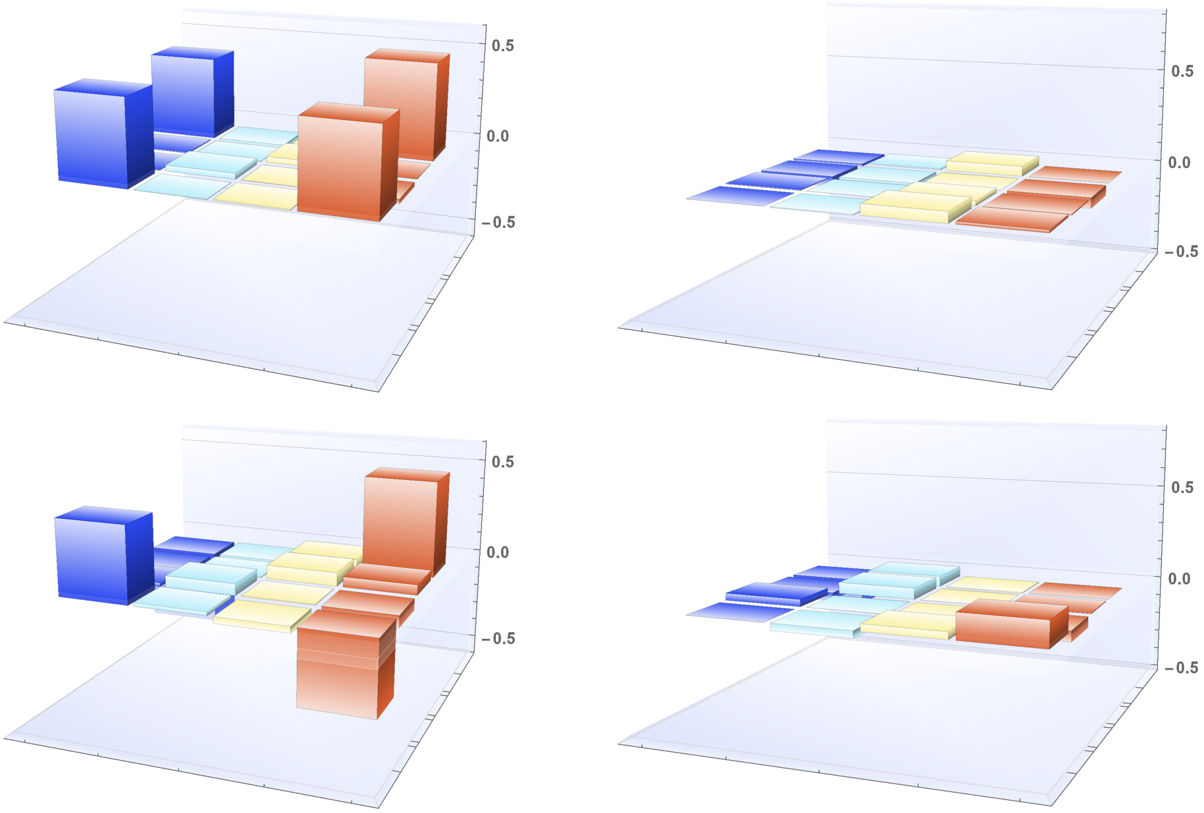

Figure S2. Examples of density matrices obtained: tomographies of $|\ell_A r_B\rangle$ and $|r_A \ell_B\rangle$ respectively (on the left side the real part, on the right side the imaginary part). As expected, they show $|\phi^+\rangle$ and $|\phi^-\rangle$ behaviors respectively.

decided by the eigenstates considered in measuring the two crossed out qubits. Given the violations of all Mermin inequalities for all the eleven possible four-, three- and two-qubit subgroupings, we will show that it is possible to quantify the quality of the entangelment that characterizes our resource.

Three-qubit groupings

Consider the case of measuring a σ_x operator on the third qubit (first path encoded) and post-select the state of the remaining 1-2-4 qubits on projecting onto the $|+\rangle_3$ eigenstate:

$$\begin{aligned}|\psi\rangle_{124} &= \frac{1}{2}(|000\rangle_{124} + |001\rangle_{124} + |110\rangle_{124} - |111\rangle_{124}) = \\ &= \frac{1}{\sqrt{2}}(|00+\rangle_{124} + |11-\rangle_{124}).\end{aligned} \tag{S19}$$

With such a state we can construct a three-qubit Mermin inequality, as it has been done in the four-qubit case. The WWZB parameter is now composed by four correlators, each for a two-setting, three-party configuration.

$$\begin{aligned}\mathrm{WWZB}_3 =& \\ &= 4\{-\mathrm{C}(\theta_{\alpha1},\phi_{\alpha1},\theta_{\beta1},\phi_{\beta1},\theta_{\gamma1},\phi_{\gamma1}) + \mathrm{C}(\theta_{\alpha1},\phi_{\alpha1},\theta_{\beta2},\phi_{\beta2},\theta_{\gamma2},\phi_{\gamma2}) + \\ &+ \mathrm{C}(\theta_{\alpha2},\phi_{\alpha2},\theta_{\beta1},\phi_{\beta1},\theta_{\gamma2},\phi_{\gamma2}) + \mathrm{C}(\theta_{\alpha2},\phi_{\alpha2},\theta_{\beta2},\phi_{\beta2},\theta_{\gamma1},\phi_{\gamma1}\}.\end{aligned} \tag{S20}$$

The bound imposed by local realistic theories is $2^3 = 8$, then $|WWZB_3| < 8$ is the inequality to beat. To construct the WWZB$_3$ parameter, we have to find the angles that maximize its value, which in this case can attain the value of 16. However, we can

Correlators	Operators	c_{teo}	c_{exp}
c_1	-JXY	-0.70	-0.44 ± 0.02
c_2	-JYY	0.70	0.66 ± 0.02
c_3	-KXY	0.70	0.72 ± 0.02
c_4	-KYY	0.70	0.51 ± 0.02

Table S.IV. Contribution of each correlator c_i to the $WWZB_3$ parameter defined in Eq.S20. In the second column, the corresponding operator to perform to the cluster state. c_{teo} is the theoretical expected value, c_{exp} is the experimental result.

exploit the same measurement of J and K performed in the four-qubit case at the cost of reaching a maximum violation of 11.31 instead of 16. The angles that allow this violation are:

$$\begin{aligned} &\theta_{\alpha 1} = \frac{3\pi}{2}, \quad \theta_{\alpha 2} = \frac{\pi}{2}, \quad \theta_{\beta 1} = \frac{\pi}{2}, \quad \theta_{\beta 2} = -\frac{\pi}{2}, \\ &\theta_{\gamma 1} = \frac{\pi}{2}, \quad \theta_{\gamma 2} = \frac{3\pi}{2}, \quad \phi_{\alpha 1} = \frac{\pi}{4}, \quad \phi_{\alpha 2} = \frac{7\pi}{4}, \\ &\phi_{\beta 1} = 0, \quad \phi_{\beta 2} = \frac{\pi}{2}, \quad \phi_{\gamma 1} = \frac{\pi}{2}, \quad \phi_{\gamma 2} = \frac{\pi}{2} \end{aligned} \tag{S21}$$

This means that the observables used in the WWZB_3 inequality for the GHZ state are:

$$\begin{aligned} r_{\alpha 1} &= -J = -(\sigma_x + \sigma_y) = \begin{pmatrix} 0 & -\frac{1-i}{\sqrt{2}} \\ -\frac{1+i}{\sqrt{2}} & 0 \end{pmatrix}, \\ r_{\beta 1} &= X = \sigma_x = \begin{pmatrix} 0 & 1 \\ 1 & 0 \end{pmatrix}, \\ r_{\gamma 1} &= Y = \sigma_y = \begin{pmatrix} 0 & -i \\ i & 0 \end{pmatrix}, \\ r_{\alpha 2} &= K = (\sigma_x - \sigma_y) = \begin{pmatrix} 0 & \frac{1+i}{\sqrt{2}} \\ \frac{1-i}{\sqrt{2}} & 0 \end{pmatrix}, \\ r_{\beta 2} &= -Y = -\sigma_y = \begin{pmatrix} 0 & i \\ -i & 0 \end{pmatrix}, \\ r_{\gamma 2} &= -Y = -\sigma_y = \begin{pmatrix} 0 & i \\ -i & 0 \end{pmatrix}. \end{aligned} \tag{S22}$$

Each term in the WWZB parameter, with the angles identified by Eq. S21, is a 3-qubit observable and contributes to the violation of the local realistic constraint according to the following definition:

$$\begin{aligned} c_1 &= \mathrm{C}(\theta_{\alpha 1}, \phi_{\alpha 1}, \theta_{\beta 1}, \phi_{\beta 1}, \theta_{\gamma 1}, \phi_{\gamma 1}) \\ c_2 &= \mathrm{C}(\theta_{\alpha 1}, \phi_{\alpha 1}, \theta_{\beta 2}, \phi_{\beta 2}, \theta_{\gamma 2}, \phi_{\gamma 2}) \\ c_3 &= \mathrm{C}(\theta_{\alpha 2}, \phi_{\alpha 2}, \theta_{\beta 1}, \phi_{\beta 1}, \theta_{\gamma 2}, \phi_{\gamma 2}) \\ c_4 &= \mathrm{C}(\theta_{\alpha 2}, \phi_{\alpha 2}, \theta_{\beta 2}, \phi_{\beta 2}, \theta_{\gamma 1}, \phi_{\gamma 1}) \end{aligned}$$

We have measured the values for each corrrelator as we did for the four-qubit case. Results are reported in Table S.IV. The WWZB_{124} parameter is:

$$WWZB_{124} = 9.32 \pm 0.19. \tag{S23}$$

where the error has been calculated by propagating the errors of the coincidences counts, assuming them to be Poissonian distributed. The value obtained is 7 standard deviations away from the bound of local realistic theories.
This procedure can be easily repeated for all the possible groupings of three qubits out of four, crossing out one qubit at a time, projecting it on a σ_x eigenvalue. It must be underlined that each of the resulting three-qubit GHZ state can always be reduced in the $|\psi\rangle_{124}$ form as in Eq.S19 by operating a simple one qubit rotation. All the results, which are reported in the main text, are inconsistent with the expectation of local realistic theories.

Two-qubit groupings

The strategy shown in the previous section can be iterated performing a projection on two qubits instead of only one, obtaining a two-qubit entangled state. This can be tested with a Mermin inequality that reduces to a simple Bell test, for all the six possible two-qubit groupings out of four: 1-2, 1-3, 1-4, 2-3, 2-4, 3-4. Generally the kind of state in which the system collapses depends on the eigenstates considered in measuring the σ_x operator on the two crossed out qubits. Besides we can always rotate the final state with a single-qubit operation, being able to use the same measures taken in the four-qubit case to maximise the violation also in a two-qubit system.

If we measure the σ_x operator on the second and third qubit (first path encoded) and consider the projection on $|+\rangle_2$ and $|+\rangle_3$ eigenstates, the state of the remaining 1-4 qubits is therefore:

$$|\psi\rangle_{14} = \frac{1}{2}(|00\rangle_{14} + |01\rangle_{14} + |10\rangle_{14} - |11\rangle_{14}) = \frac{1}{\sqrt{2}}(|0+\rangle_{14} + |1-\rangle_{14}). \tag{S24}$$

With this kind of state we can build a two-qubit Mermin inequality as it has been done in the four- and three-qubit case. The $WWZB_2$ parameter is now composed by four correlators, each for a two-setting, two-party configuration, as in the Bell inequality:

$$\begin{aligned} \mathrm{WWZB}_2 = & \\ & = 2\{-\mathrm{C}(\theta_{\alpha 1}, \phi_{\alpha 1}, \theta_{\beta 1}, \phi_{\beta 1}) + \mathrm{C}(\theta_{\alpha 1}, \phi_{\alpha 1}, \theta_{\beta 2}, \phi_{\beta 2}) + \\ & + \mathrm{C}(\theta_{\alpha 2}, \phi_{\alpha 2}, \theta_{\beta 1}, \phi_{\beta 1}) + \mathrm{C}(\theta_{\alpha 2}, \phi_{\alpha 2}, \theta_{\beta 2}, \phi_{\beta 2})\}. \end{aligned} \tag{S25}$$

The bound imposed by local realistic theories is $2^2 = 4$, then $|WWZB_2| < 4$ is the inequality to beat. We proceed as above to find the angles that maximize its value, in this case $4\sqrt{2}$. The angles that allow this violation are:

$$\begin{aligned} &\theta_{\alpha 1} = \frac{\pi}{2}, \quad \theta_{\alpha 2} = \frac{3\pi}{2}, \quad \theta_{\beta 1} = \frac{\pi}{2}, \quad \theta_{\beta 2} = \frac{\pi}{2}, \\ &\phi_{\alpha 1} = \frac{\pi}{4}, \quad \phi_{\alpha 2} = \frac{3\pi}{4}, \quad \phi_{\beta 1} = \frac{\pi}{2}, \quad \phi_{\beta 2} = 0. \end{aligned} \tag{S26}$$

The observables used in the WWZB_2 inequality are:

$$\begin{aligned} r_{\alpha 1} &= J = \begin{pmatrix} 0 & \frac{1-i}{\sqrt{2}} \\ \frac{1+i}{\sqrt{2}} & 0 \end{pmatrix}, \\ r_{\beta 1} &= Y = \begin{pmatrix} 0 & -i \\ i & 0 \end{pmatrix} \\ r_{\alpha 2} &= K = \begin{pmatrix} 0 & \frac{1+i}{\sqrt{2}} \\ \frac{1-i}{\sqrt{2}} & 0 \end{pmatrix}, \\ r_{\beta 2} &= X = \begin{pmatrix} 0 & 1 \\ 1 & 0 \end{pmatrix} \end{aligned} \tag{S27}$$

Each term in the WWZB parameter, with the angles defined in S26, is a two-qubit observable and contributes to the violation as indicated by c_{teo}, while experimental results are indicated by c_{exp} in Table S.V. The WWZB_{14} parameter is:

$$WWZB_{14} = 4.55 \pm 0.13. \tag{S28}$$

The value obtained is 4 standard deviations away from the bound of local realistic theories. We have followed the same procedure for the remaining cases of two-qubit subgroupings. The results are summarised in the main text.

Assessment of correlations quality

Given the violations of all the Mermin inequalities for all the eleven possible four-, three- and two-qubit subgroupings, we can reconstruct the strength of the corelations between the cluster components, i.e. the quality of the entangelment shared among the four qubits. This can be done by comparing the results of the WWZB parameters obtained, with the ones of a theoretical specular resource corrupted by noise.

Correlators	Operation	c_{teo}	c_{exp}
c_1	JY	-0.70	-0.39 ± 0.02
c_2	JX	0.70	0.49 ± 0.02
c_3	KY	0.70	0.77 ± 0.02
c_4	KX	0.70	0.63 ± 0.02

Table S.V. Contribution of each correlator c_i to the $WWZB_2$ parameter defined in Eq.S25. In the second column, the corresponding operator to perform to the cluster state. c_{teo} is the theoretical expected value, c_{exp} is the experimental result.

Noisy entangling gates

As shown in Ref.[4] and Ref.[5], a for-qubit linear cluster state can be obtained applying a chain of three C-phase gates on an initial $|++++\rangle_{1234}$ state. If it is prepared in states $|0000\rangle_{1234}$ or $|1111\rangle_{1234}$, no entanglement will build up. The three C-phase gates actually build the entanglement links between the cluster's qubits, according to the scheme in Fig. S3:

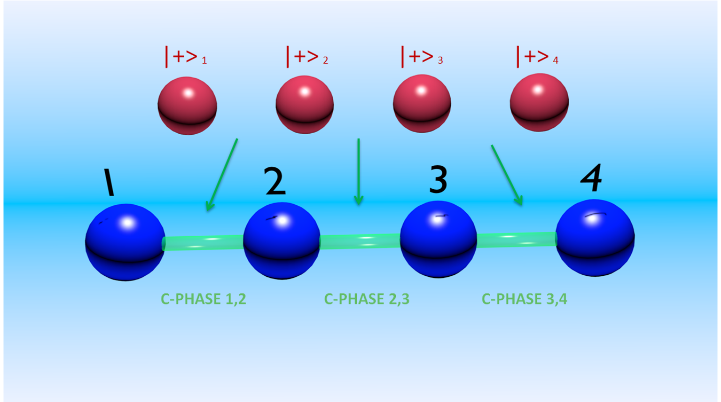

Figure S3. Building of entanglement on an initial separable $|++++\rangle_{1234}$ state. A linear cluster state can be easily obtained by chaining three C-phase gates on the four initial qubits: C-PHASE$_{1,2}$, C-PHASE$_{2,3}$, C-PHASE$_{3,4}$.

The application of a chain of the three operators on the prepared state produces the following linear cluster state:

$$|\Gamma_4\rangle = \frac{1}{2}(|0000\rangle_{1234} + |0011\rangle_{1234} + |1100\rangle_{1234} + |1111\rangle_{1234}), \tag{S29}$$

that can be reduced to our linear cluster state by simply applying the unitary rotation:

$$U = (\sigma_x H \otimes -\sigma_x \otimes \sigma_x \otimes \sigma_z H).$$

Here H is the single qubit Hadamard gate. To account for imperfections, we apply to the initial state a probabilistic C-phase gates, i.e. gates that acts with a probability p as a C-phase and fail completely with a probability $(1-p)$. We can write the effect of these gates as a Kraus maps:

$$\begin{aligned} M_{12}(\hat{\rho}) &= p_1\,(\hat{O}_{12}\,\hat{\rho}\,\hat{O}_{12}^\dagger) + (1-p_1)\hat{\rho} \\ M_{23}(\hat{\rho}) &= p_2\,(\hat{O}_{23}\,\hat{\rho}\,\hat{O}_{23}^\dagger) + (1-p_2)\hat{\rho} \\ M_{34}(\hat{\rho}) &= p_3\,(\hat{O}_{34}\,\hat{\rho}\,\hat{O}_{34}^\dagger) + (1-p_3)\hat{\rho} \end{aligned} \tag{S30}$$

When these three three maps act on the initial $\hat{\rho}_{in} = (|++++\rangle_{1234})({}_{1234}\langle++++|)$ state in a chain, we obtain the state:

$$\hat{\rho}_{fin}(p_1, p_2, p_3) = M_{34}(M_{23}(M_{12}(\hat{\rho}_{in}))). \tag{S31}$$

In this way, after applying the unitary U, we have expressed the linear cluster state, Eq. S8, as a function of the probabilities

that describe the action of the probabilistc C-phase gates. From the analitic expression of the state we can calculate the expected values of the WWZB parameters.

$$\begin{cases} WWZB_{1234} = 16\sqrt{2}\,p_1\,p_3; \\ WWZB_{124} = 8\sqrt{2}\,p_1\,p_2\,p_3; \\ WWZB_{123} = 4[\sqrt{2}\,p_1\,(1-2\,p_2)(1-2\,p_3) - \sqrt{2}(p_1 - 2\,p_1\,p_3)]; \\ WWZB_{134} = 4\,[\frac{p_1 p_3}{\sqrt{2}} + p_1\,p_3(2\,p_2 - 1) + \frac{p_1 p_3(2p_2-1)}{\sqrt{2}} - p_3(-4+3p_1 \\ +4p_2 - 4p_1 p_2)]; \\ WWZB_{234} = 4\,[p_3(1+\frac{1}{\sqrt{2}}) + p_3(2p_2-1) + \frac{p_3(2p_2-1)}{\sqrt{2}}]; \\ WWZB_{14} = 4\,\sqrt{2}\,p_1\,[1 - p_3 + p_2(2p_3 - 1)]; \\ WWZB_{13} = 4\,\sqrt{2}\,p_1\,p_2\,p_3; \\ WWZB_{23} = 4\,\sqrt{2}\,p_2\,p_3; \\ WWZB_{24} = 4\,\sqrt{2}\,[1 - p_3 + p_2(2p_3 - 1)]; \\ WWZB_{12} = 4\,\sqrt{2}\,p_1; \\ WWZB_{34} = 4\,\sqrt{2}\,p_1 p_3(2p_2 - 1); \end{cases} \tag{S32}$$

Now we can find the values of p_1, p_2, p_3 that reproduce the WWZBs obtained in the experiment, by a maximum likelihood fitting:

$$p_1^* = 0.975 \pm 0.024 \qquad p_2^* = 0.992 \pm 0.010 \qquad p_3^* = 0.842 \pm 0.022, \tag{S33}$$

having imposed as constraints that each of the WWZB parameters is Gaussian distributed assuming its error as standard deviation.

Dephasing channel

An analogous method of evaluating the quality of the cluster resource is comparing it with a resource damaged by a dephasing channel acting on each component of the system. This is an alternative perspective that focalizes on the resource from the point of view of the single qubits quality rather than the quality of a two-qubit link.The dephasing channel is a Kraus operator that acts as follows:

$$\hat{\rho} \quad \rightarrow \quad \hat{\rho}' = \varepsilon(\hat{\rho}) = \sum_{k=1}^{3} E_k \hat{\rho} E_k^{\dagger} \tag{S34}$$

where the Kraus operators E_k are:

$$E_1 = \sqrt{p}\begin{pmatrix} 1 & 0 \\ 0 & 1 \end{pmatrix} = \sqrt{p}\,\hat{\mathbf{I}}, \tag{S35}$$

$$E_2 = \sqrt{1-p}\begin{pmatrix} 1 & 0 \\ 0 & 0 \end{pmatrix} = \sqrt{1-p}|0\rangle\langle 0|, \tag{S36}$$

$$E_3 = \sqrt{1-p}\begin{pmatrix} 0 & 0 \\ 0 & 1 \end{pmatrix} = \sqrt{1-p}|1\rangle\langle 1|, \tag{S37}$$

so that the dephased state is:

$$\hat{\rho}' = p\,\hat{\rho} + (1-p)(\hat{\rho}_{00} + \hat{\rho}_{11}) = p\,\hat{\rho} + (1-p)\mathrm{diag}(\hat{\rho}). \tag{S38}$$

If p=1 the state is left unchanged. If p=0, only the diagonal terms of the density matrix $\hat{\rho}$ survive, i.e. the state is completely mixed, having lost all the correlation terms.

In our case the overall Kraus map, acting on each of the components of cluster state, is the Kronecker product of four dephasing channels as in S34:

$$\varepsilon_{tot}(\hat{\rho}_{cluster}) = \varepsilon_1(p_1)\otimes\varepsilon_2(p_2)\otimes\varepsilon_3(p_3)\otimes\varepsilon_4(p_4), \tag{S39}$$

where $\varepsilon_i(p_i)$ represents the ith dephasing channel acting on the ith qubit. This overall map is still a Kraus Map, composed by $3^4 = 81$ Kraus operators (the product of the sum over three Kraus operators each qubit). Applying this overall map we can therefore obtain the dephased cluster state as a function of (p_1, p_2, p_3, p_4) (i.e. the non-dephasing probabilities for each qubit). Proceeding in an analogous way as in the case of noisy gate, we can determine the degree of dephasing characterizing our resource. Again we can calculate all the eleven WWZB parameters, now functions of (p_1, p_2, p_3, p_4), using exactly the same procedure described in the previous section, that means operating the same measures to build the same correlators.

$$\begin{cases} WWZB_{1234} = 16\sqrt{2}\,p_1\,p_2\,p_3\,p_4; \\ WWZB_{124} = 8\sqrt{2}\,p_1\,p_2\,p_3\,p_4; \\ WWZB_{123} = 8\sqrt{2}\,p_1\,p_2\,p_3\,p_4; \\ WWZB_{134} = 4\,(2p_3\,p_4 + \sqrt{2}\,p_1\,p_2\,p_3\,p_4); \\ WWZB_{234} = 4\,(2p_3\,p_4 + \sqrt{2}\,p_1\,p_2\,p_3\,p_4); \\ WWZB_{14} = 2\sqrt{2}\,p_1 p_2\,[1 + p_3\,p_4]; \\ WWZB_{13} = 2\sqrt{2}\,p_1 p_2\,[1 + p_3\,p_4]; \\ WWZB_{23} = 2\sqrt{2}\,p_1 p_2\,[1 + p_3\,p_4]; \\ WWZB_{24} = 2\sqrt{2}\,p_1 p_2\,[1 + p_3\,p_4]; \\ WWZB_{12} = 4\sqrt{2}\,p_1\,p_2; \\ WWZB_{34} = 4\sqrt{2}\,p_3 p_4; \end{cases} \tag{S40}$$

Note that, applying the overall map on the original undisturbed density matrix of the cluster state, with $(p_1, p_2, p_3, p_4) = (1,1,1,0)$ we kill not only the correlations between the 4th qubit and the others, but also the correlations of the 3rd and the others. In other words we are damaging the correlations between the two couples $1^{st} - 2^{nd}$ and $3^{rd} - 4^{th}$ as we could have done by imposing $(p_1, p_2, p_3, p_4) = (1,1,0,1)$ or $(1,1,0,0)$. The same can be observed damaging the qubit 1st and 2nd. The loss of all correlations can be therefore observed when at least three qubits are ruined with a $p_i = 0$: $(1,0,0,0)$, $(0,1,0,0)$, $(0,0,1,0)$, $(0,0,0,1)$ or $(0,0,0,0)$. This symmetry can be explained by the fact that the entangling gate that actually builds the cluster state, starting from a HE state, is a C-phase: this relies intrinsically on the action of a phase that, if destroyed, destroys the nature of the cluster itself. It is worth noting that some WWZBs have the same analytic expression. For example $WWZB_{124}$ and $WWZB_{123}$ or $WWZB_{14}$, $WWZB_{13}$, $WWZB_{24}$ and $WWZB_{23}$. Furthermore it must be highlighted that the couple p_1 and p_2 and the couple p_3 and p_4 can never be determined singularly, because they always occur as a product. These two characteristics seem reasonable since the symmetries of the channel combined with the ones of the state discussed above.

We can find the values of p_1, p_2, p_3, p_4 that reproduce the WWZBs obtained. The strategy is exactly the same as in the previous case: finding $(p_1^*, p_2^*, p_3^*, p_4^*)$ that minimize the difference between each of the WWZBs espressed above and the corresponding WWZB experimentally obtained. The results are:

$$p_1^* = 0.954 \pm 0.038 p_2^* = 0.957 \pm 0.038$$
$$p_3^* = 0.949 \pm 0.045 p_4^* = 0.940 \pm 0.045.$$

having imposed as constraints that each of the WWZB parameters is Gaussian distributed assuming its error as standard deviation.

References

1. Żukowski, M. & Brukner, Č. Bell's Theorem for General N-Qubit States. *Phys. Rev. Lett.* **88**, 210401 (2002).
2. Werner R. F. & Wolf, M. M. All-multipartite Bell-correlation inequalities for two dichotomic observables per site. *Phys. Rev. A* **64**, 032112 (2001).

3. Collins, D.*et al.*, Bell-Type Inequalities to Detect True n-Body Nonseparability. *Phys. Rev. Lett.* **88**, 170405 (2002).

4. Raussendorf R. & Briegel, H. J. A one-way quantum computer. *Phys. Rev. Lett.* **86**, 5188 (2001).

5. Briegel H. J. & Raussendorf, R. Persistent Entanglement in Arrays of Interacting Particles. *Phys. Rev. Lett.* **86**, 910 (2001).
